# Antibacterial Effect of Chitosan–Gold Nanoparticles and Computational Modeling of the Interaction between Chitosan and a Lipid Bilayer Model

**DOI:** 10.3390/nano10122340

**Published:** 2020-11-25

**Authors:** M. G. Fuster, M. G. Montalbán, G. Carissimi, B. Lima, G. E. Feresin, M. Cano, J. J. Giner-Casares, J. J. López-Cascales, R. D. Enriz, G. Víllora

**Affiliations:** 1 Chemical Engineering Department, Faculty of Chemistry, University of Murcia, 30100 Murcia, Spain; marta.g.f@um.es (M.G.F.); guzmanaugusto.carissimin@um.es (G.C.); gvillora@um.es (G.V.); 2 Instituto de Biotecnología, Universidad Nacional de San Juan, Av. Libertador General San Martín 1109 (O), San Juan CP 5400, Argentina; blima@unsj.edu.ar (B.L.); gferesin@unsj.edu.ar (G.E.F.); 3 CONICET (Consejo Nacional de Ciencia y Tecnología), CABA, Buenos Aires C1405DJR, Argentina; 4 Departamento de Química Física y Termodinámica Aplicada, Instituto Universitario de Nanoquímica (IUNAN), Facultad de Ciencias, Universidad de Córdoba, Campus de Rabanales, Ed. Marie Curie, E-14071 Córdoba, Spain; q82calum@uco.es (M.C.); jjginer@uco.es (J.J.G.-C.); 5 Departamento Ingeniería Química y Ambiental, Campus Alfonso XIII, Universidad Politécnica de Cartagena, Aulario C, Cartagena, 30203 Murcia, Spain; javier.lopez@upct.es; 6 Facultad de Química, Bioquímica y Farmacia, Instituto Multidisciplinario de Investigaciones Biológicas (IMIBIO-SL), CONICET, Universidad Nacional de San Luis, Ejército de los Andes 950, San Luis 5700, Argentina; denriz@unsl.edu.ar

**Keywords:** chitosan–gold nanoparticle, antimicrobial activity, computational model

## Abstract

Pathogenic bacteria have the ability to develop antibiotic resistance mechanisms. Their action consists mainly in the production of bacterial enzymes that inactivate antibiotics or the appearance of modifications that prevent the arrival of the drug at the target point or the alteration of the target point itself, becoming a growing problem for health systems. Chitosan–gold nanoparticles (Cs-AuNPs) have been shown as effective bactericidal materials avoiding damage to human cells. In this work, Cs-AuNPs were synthesized using chitosan as the reducing agent, and a systematic analysis of the influence of the synthesis parameters on the size and zeta potential of the Cs-AuNPs and their UV-vis spectra was carried out. We used a simulation model to characterize the interaction of chitosan with bacterial membranes, using a symmetric charged bilayer and two different chitosan models with different degrees of the chitosan amine protonation as a function of pH, with the aim to elucidate the antibacterial mechanism involving the cell wall disruption. The Cs-AuNP antibacterial activity was evaluated to check the simulation model.

## 1. Introduction

The global increase in outbreaks and mortality rates associated with multi drug resistant bacteria has become an important factor in the fight against infectious diseases [1]. The bacterial resistance to antibiotics is a great problem because these infections are sometimes impossible to treat, causing the deaths of many people around the world [2]. According to the World Health Organization, at least 700,000 people die every year due to drug-resistant diseases. Among the resistant bacteria, *Escherichia coli* (Gram-negative) and *Staphylococcus aureus* (Gram-positive) are the most common Gram-negative and Gram-positive bacterial pathogens, respectively, causing a diverse range of clinical diseases such as pneumonia and chronic infections [3,4]. The resistance of these bacteria to various antibiotics has promoted an urgent search for effective antibacterial agents with new mechanisms to treat infections. 

During the last decade, nanoparticles made of different materials and with diameters of less than 200 nm have been widely studied for application in medicine [5], including as antibacterial agents [6,7]. Noble metal nanoparticles, especially gold nanoparticles (AuNPs), have attracted the most attention because of their interesting and unique properties, which makes them suitable for applications in biomedical sciences [8,9], especially due to their biocompatibility and non-toxicity [6,10,11]. Cui et al. [12] found that AuNPs exert their antibacterial action mainly by two ways: one is to change membrane potential and inhibit ATP synthase activities to decrease the ATP level, indicating a general decline in metabolism; the other is to inhibit the subunit of ribosome for tRNA binding, indicating a collapse of the biological process. Among all the physical and chemical methods for producing AuNPs described in the literature, chemical reduction, in which a gold precursor (usually Au(III) in the form of HAuCl_4_ or AuCl_4_^−^) is reduced by a reducing agent, is the most efficient because of its simplicity [13]. However, harsh chemical reducing agents such as borohydride have been used for this process, and large volumes of organic solvents, such as *n*-hexane, chloroform or *N,N*-dimethylformamide, and also high temperatures are needed for the development of the AuNPs [8]. This inevitably creates serious environmental issues for the industrial production of AuNPs and, at the same time, restricts their application in biomedical fields. Recently, green synthesis methods for the synthesis of AuNPs have attracted attention. Some of the key parameters involved in such green synthetic strategies are the use of non-toxic chemicals, environmentally benign solvents or renewable materials [14].

In addition, during the synthesis process, a stabilizing agent is required to obtain a monodisperse solution of AuNPs because they tend to aggregate through van der Waals interactions, leading to Ostwald ripening [13]. The absence of a stabilizing coating against the high ionic strength and organic content found in physiological conditions would hinder their application for medical purposes. The use of specific polymers as stabilizing agents in the production of metallic nanoparticles has been shown to control particle size through the surface modification of AuNPs [15]. Currently, according to the principles of the green synthesis or biosynthesis of AuNPs, the use of materials from natural sources is attracting great attention. Due to their availability, relatively low cost, and multifunctionality, polysaccharides such as chitosan [6,13], starch [16] and alginate [17] are the natural polymers of greatest interest for use as stabilizers in the synthesis of AuNPs. Among these, chitosan stands out because it can act simultaneously as a stabilizing and reducing agent, mainly due to abundant presence of amino and hydroxyl groups [18,19].

Chitosan is a cationic biopolymer obtained by total or partial *N*-deacetylation of the natural polysaccharide chitin. It consists of D-glucosamine and *N*-acetyl-D-glucosamine units connected through β-1,4 glycosidic linkages, the ratio between both units being known as the degree of deacetylation. The positive charge density of chitosan, which is provided by the –NH^3+^ groups, provides antibacterial and antimycotic properties because of its interaction with the negatively charged cell membranes of a considerable number of Gram-positive and Gram-negative bacteria. The antimicrobial activity of chitosan largely depends on the type of chitosan, the degree of deacetylation and the molecular weight, as well as on other parameters that depend on the conditions of the medium, such as pH, ionic strength and the presence of solutes susceptible to react with the biopolymer through electrostatic interactions and/or covalent bonds, which could block or screen the reactivity of the active –NH^3+^ group. In recent studies [20,21], chitosan is considered a bactericidal (kills live bacteria or some fraction) or bacteriostatic (hinders the growth of bacteria but does not imply whether or not bacteria are killed), often without distinction between activities [22].

Three mechanisms of action have been proposed for the activity of chitosan against microorganisms. The most acceptable is the one that proposes the interaction between positively charged chitosan molecules and negatively charged microbial cell membranes. In this model, the interaction is mediated by the electrostatic forces between the protonated NH^3+^ groups and the negative residues, presumably competing with Ca^2+^ by electronegative sites on the membrane surface [23]. This electrostatic interaction produces changes in the permeability properties of the membrane wall, which cause osmotic imbalances and inhibit the growth of bacteria and also hydrolysis of peptidoglycans in the wall of the microorganism, which leads to the leakage of intracellular electrolytes such as potassium ions and other low molecular weight protein components [24]. Another mechanism proposes that the bond of chitosan with microbial DNA causes the inhibition of mRNA and protein synthesis by penetrating chitosan into the nuclei of the bacteria through the cell wall, composed of crosslinked murein multilayers, and reaching the plasma membrane [25]. Finally, the third mechanism consists in the chelation of metals with chitosan [26], which has excellent metal-binding capacities, and linkage to essential nutrients to microbial growth. Amine groups in the chitosan molecules are responsible for the uptake of metal cations by chelation. In general, such a mechanism is more efficient at high pH, where positive ions are bounded to chitosan, since the amine groups are unprotonated and the electron pair on the amine nitrogen is available for donation to metal ions.

The combination of AuNPs and chitosan in the same particle seems promising because, as mentioned above, the positively charged chitosan potentiates interactions with bacteria, allowing the positively charged AuNPs to better disrupt the anionic bacterial cell membrane. In this way, chitosan enhances the biocompatibility and antibacterial activity of AuNPs [6,27]. The physical and chemical properties of chitosan–gold nanoparticles (Cs-AuNPs) depend on their size, shape, structure and stability, which can be controlled by varying the experimental parameters during synthesis. Analyzing the influence of these parameters is crucial for designing a scalable synthesis process of antimicrobial AuNPs which can be exploited for therapeutic applications. 

On the other hand, recently, there is a consensus in scientific areas that membranes also constitute a biochemical platform for cell signaling, with proteins interacting with membranes, as well as pathogens that take advantage of intrinsic differences between cell membranes. Nanoparticles have also been shown to interact extensively with membranes and can be taken by cells that use pathways similar to those of pathogens. In this context, molecular dynamics simulations have emerged as one of the methodologies to characterize interactions between cell membranes and nanoparticles, as this approach offers the possibility to investigate the behavior of matter directly at the atomistic level under highly controlled conditions [28,29]. In particular, molecular simulations have been widely used to clarify which mechanisms drive the nanoparticle absorption process in the cell, focusing on membrane vesiculation, endocytic routes, or passive permeation processes [30]. Although Melby et al. [31] and Irudayanathan et al. [32] have performed molecular dynamics simulations studying the interactions of the bacterial membrane systems with two types of anionic nanoparticles, most of the computational studies have been restricted to rigid ligand docking approaches, with no consideration for the bacterial phospholipid membrane or the solvent environment. This study is aimed to develop simulations by molecular dynamics to characterize the interaction of chitosan with bacterial membrane, using a symmetric charged bilayer and two different chitosan models with different degrees of the chitosan amine protonation as a function of pH, with the aim to elucidate the antibacterial mechanism involving the cell wall disruption. The antimicrobial activity was assessed to check the model and to be correlated with the physical properties of the Cs-AuNPs (size, polydispersity, zeta potential and electrophoretic mobility). In addition, the influence of the operating variables such as chitosan molecular weight, chitosan and gold concentrations or reaction solvent used were analyzed in terms of the physical properties of the Cs-AuNPs. Furthermore, the estimated values of the nanoparticle diameter based on the UV-vis spectra and the values obtained from dynamic light scattering (DLS) measurements and from TEM have been compared to determinate the core size and the total size of the core-shell particle, due to their importance for biomedical applications. 

## 2. Materials and Methods

### 2.1. Materials

Very low molecular weight (VLM_w_) chitosan (molecular weight of 30 kDa, ≥90% deacetylation degree) was purchased from Glentham Life Sciences (Corsham, UK). Low molecular weight (LM_W_) chitosan (molecular weight of 19−250 kDa, deacetylation degree of 75−85%), medium molecular weight (MM_W_) chitosan (molecular weight of 310 kDa, deacetylation degree of 75−85%) and high molecular weight (HM_W_) chitosan (molecular weight of 375 kDa, deacetylation degree of >75%) were purchased from Sigma-Aldrich (Madrid, Spain). Glacial acetic acid (99.7% purity) used for the preparation of the chitosan solution, hydrogen tetrachloroaurate (III) trihydrate (HAuCl_4_ ·3H_2_O) (≥99.9% trace metals basis), methanol (>99.9% purity) and isopropanol (>99.9% purity) were also procured from Sigma-Aldrich (Madrid, Spain). Ultrapure distilled water was obtained from a Merck Millipore Milli-Q purifier system (Darmstadt, Germany).

### 2.2. Chitosan–Gold Nanoparticles Preparation

The Cs-AuNPs were prepared following the protocol described elsewhere with slight modifications [33]. All glassware was carefully cleaned with *aqua regia* and then rinsed thoroughly with water before use. Chitosan solutions with concentrations of 0.1 or 0.4 % (*w*/*v*) (depending on the experiment) were prepared using 1% *v*/*v* aqueous acetic acid solutions under magnetic stirring for 24 h, until the solution was clearly homogeneous. After filtration through a 0.22 µm filter, 30 mL of the solution were poured into a glass reactor previously heated to 80 °C. A stock aqueous solution of 20 mg/mL of HAuCl_4_ was freshly prepared. A predetermined volume of the HAuCl_4_ was added to the reactor, adjusting the concentration of gold to 1.3·10^−4^M or 2.6·10^−4^M (depending on the experiment) in order to achieve chitosan:gold molar ratios of 20:1, 40:1, 80:1 and 160:1. The mixtures were continuously stirred magnetically at 80 °C for 3 h. In order to keep a constant volume in the reaction, a water refluxing system at 4 °C was used. The color of the reaction product varied from purplish to wine or pale red. After the product reached room temperature, the Cs-AuNPs were characterized. Methanol and isopropanol were used to evaluate the influence of the reaction medium. In these cases, the final volume in the reaction was also 30 mL: 15 mL of (acetic acid + chitosan) and 15 mL of methanol or isopropanol. Table 1 shows a summary of the experiments performed and their experimental parameters.

### 2.3. Characterization of the Chitosan-Gold Nanoparticles

#### 2.3.1. Dynamic Light Scattering (DLS)

After the reaction was completed, the mean hydrodynamic diameter (expressed as Z-average), the polydispersity index (PdI), the zeta potential and the electrophoretic mobility of the Cs-AuNPs were determined by DLS using a Zetasizer Nano ZS instrument (Malvern Instruments Ltd., Worcestershire, UK). All measurements were obtained in the same medium as the reaction at 25 °C and at a 173° angle relative to the source. All measurements were performed in triplicate and values were expressed as mean ± SD. The values of the surface charge density have been calculated for all the Cs-AuNPs using the values of the radius and the zeta potential. The calculation method, the results obtained (Table S1) and the parameters used (Table S2) have been reported in the Supplementary Materials.

#### 2.3.2. UV-Vis Spectroscopy

UV-vis absorbance spectra of the synthesized Cs-AuNPs were recorded on a Thermo Spectronic Heλios α spectrophotometer (Thermo Fisher Scientific, Waltham, MA, USA) in the wavelength range of 750−450 nm and the diameter, *d*, of the AuNPs obtained in each experiment was estimated as reported in the Supplementary Materials.

#### 2.3.3. Transmission Electron Microscopy (TEM)

Samples for transmission electron microscopy (TEM) were prepared by drying a dispersion of the particles in ambient conditions on 200 mesh copper grids coated with Formvar/carbon films. TEM images were obtained using a JEOL JEM 1400 TEM microscope(Peabody, MA, USA), operated at an accelerating voltage of 80 kV. The size of the AuNPs was quantified by ImageJ software (National Institute of Health (NIH), USA). TEM images of the Cs-AuNPs (experiment 4) with and without staining using uranyl acetate have been taken to obtain additional information on the morphology of the coating layer of chitosan

#### 2.3.4. Antibacterial Assay

Microorganisms: three American Type Culture Collection (ATCC) reference strains were selected, two Gram-positive bacterial strain methicillin-sensitive *Staphylococcus aureus* ATCC 29213 (MSSA), methicillin-resistant *Staphylococcus aureus* ATCC 43300 (MRSA), and Gram-negative *Escherichia coli* ATCC 25922 (EC), as well as a clinical isolate of *Escherichia coli* 11046 (CI-EC) provided by the Microbiology Laboratory of the Public Hospital Dr. Marcial Quiroga (San Juan, Argentina).

The minimum inhibitory concentration (MIC) of Cs-AuNPs and the reference antibiotic Cefotaxime (Argentia^®^, Buenos Aires, Argentina) was measured using broth microdilution techniques, as recommended by Clinical and Laboratory Standards Institute (CLSI) guidelines [34]. Stock solutions of Cs-AuNPs were added to the medium to obtain final concentrations ranging from 100 to 12.5 µg/mL. All tests were performed in Mueller–Hinton broth (MHB), and cultures of each strain were prepared overnight. The inoculum of each bacterium was adjusted to 5 × 10^5^ cells with colony forming units (CFU)/mL in a spectrophotometer with sterile physiological solutions to give a final density of 0.5 on the Mc Farland scale. A volume of 100 μL of inoculum suspension was added to each well, except the sterile control to which sterile water was added. The plates were incubated at 37 °C, and data were recorded at 6, 9, 12, 15, 18 and 24 h. The absorbances at 620 nm were determined in a Multiskan FC Microplate Photometer (Thermo Scientific, Waltham, MA, USA). The minimum bactericidal concentration (MBC) test was performed via inoculation of MIC broth (5 µL) on culture plates containing nutrient agar. The MIC and MBC values were defined as the lowest Cs-AuNP concentrations showing no bacterial growth after the incubation time. Tests were carried out in triplicate. MIC and MBC values were expressed in µg/mL. 

### 2.4. Computer Simulation Parameters

GROMACS package 4.5.3 was used to calculate the molecular dynamic trajectories. The integration time step was 2 fs. Van der Waals forces were simulated using the Lennard Jones potential (LJ), and the electrostatic interactions were calculated with the particle mesh ewald (PME) method [35,36]. In both cases, a cut-off of 1 nm was applied. The bond lengths were restrained using LINCS [37]. All simulations were carried out in an NPT ensemble at 350 K and 1 atm, coupled to weak temperature and pressure bath algorithms [38], with time constants of 0.1 ps and 1 ps for temperature and pressure, respectively. The Ryckaert–Bellemans potential [39] was used in all torsions along the aliphatic chains, to better reproduce the cis-trans transitions. The water model considered in all simulations was Simple Point Charge (SPC) [40]. Due to the anisotropy of the membrane along the X-axis, all the simulations were carried out using a semi-isotropic pressure algorithm coupling bath pressure.

GROMOS force field [41] was used as a basis for all our simulations. This force field has been widely used in the simulation of lipid bilayers and monolayers, and their interactions with other penetrant molecules such as probes, anesthetics and peptides [42,43]. The LJ parameters, bond angles, dihedral interactions and force constants used in these simulations for DPPC and DPPS were the same as those proposed by [44] and which have been verified in previous simulations of lipid bilayers [45,46,47].

#### 2.4.1. Lipid Bilayer Model

The bacterial membrane was simulated using a symmetric charged bilayer composed of 96 DPPS– (dipalmitoylphosphatidylserine) and 192 DPPC (dipalmitoylphosphatidylCholine), in which DPPS– represents 20% of the molecular fraction in the lipid bilayer. That model resembles the bacterial charged cell membrane, as has been described elsewhere [48]. The simulation temperature was 350K to ensure that both DPPC and DPPS– were in their respective liquid crystalline state, with possess transition temperatures of 314 K [49] and 326 K [50], respectively. This temperature (350 K) is above the transition temperature of all the binary bilayers formed by DPPC/DPPS–, as deduced from its experimental phase diagram [51].

#### 2.4.2. Chitosan Model

**Figure 1:**
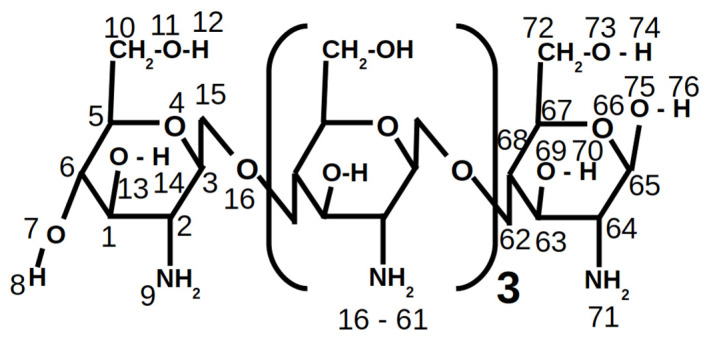
Chitosan model used in our simulations, in which the atom numeration used for its atomic charge distribution is included.

Figure 1 shows a scheme of the chitosan model used in our simulations which was composed of five repetitive units. In this regard, two different chitosan models were simulated, which we called CT+1 (with charge +1) and CT+5 (with charge +5), which represented different degrees of the chitosan amine protonation as a function of pH. Table S3 of the Supplementary Materials shows the atomic charge distribution calculated for both chitosan models using the semi-empirical complete neglect of differential overlap (CNDO) method [52] included in the Hyperchem package [53].

Chitosan aggregates were simulated considering 18 molecules of CT+1 or CT+5, respectively. Unfortunately, the AuNPs could not be considered in our simulations because their particle size exceeded the dimensions that are feasible in a MD simulation with atomic detail.

#### 2.4.3. Simulation Box

Two simulation boxes were generated to study the interaction between chitosan and a lipid bilayer model, as a function of the chitosan charge.

- System in the presence of CT+1

In this case, we consider that the total charge of each chitosan molecule is +1 (hereafter called CT+1). Thus, the simulation box in this case was constituted by 96 DPPS + 192 DPPC + 18 (CT+1) + 96 Na+ + 18 Cl^−^ + 12,546 SPC water molecules, which totaled 53,538 atoms. The dimensions of the periodical computational box after minimizing the potential energy of the system were the following: box-X = 8.65 nm, box-Y = 8.58 nm and box-Z = 10.48 nm.

- System in the presence of CT+5

In this second case, we consider that each chitosan molecule carries 5 positive charges (hereafter called CT+5), which corresponds to the protonation of all the amine groups of the chitosan model described above. Thus, in this case, it is formed by 96 DPPS + 192 DPPC+ 18 CT+5 + 96 Na+ + 90 Cl^−^ + 12546 SPC water molecules, totaling 53,592 atoms. The dimensions of the periodical computational box after minimizing the total energy of the system were as follows: box-X = 9.21 nm, box-Y = 9.13 nm and box-Z = 9.51 nm.

### 2.5. Statistical Analysis

All data are presented as the mean ± standard deviation (SD) calculated from at least three independent samples per condition using GraphPad Prism 8.0.1 software (GraphPad Software, San Diego, CA, USA). As normality (Kolmogorov–Smirnov, *p* > 0.05) and homoscedasticity (Levene, *p* > 0.05) were met, the statistical significance was determined using the parametric tests of Tukey (*p* < 0.05) and ANOVA (*p* < 0.05) for the comparisons of two or more groups, respectively.

## 3. Results and Discussion

It has previously been demonstrated that AuNPs can be biosynthesized using chitosan as both reducing and stabilizing agent [18,54]. In addition, previous studies have shown that varying synthesis parameters such as the chitosan molecular weight [55], concentration of chitosan [55] and Au(III) ions [56], or reaction solvent [57], enables chitosan-stabilized AuNPs of various shapes and sizes to be obtained. Following these approaches, we prepared Cs-AuNPs by direct Au(III) ion reduction with chitosan solutions. To study the influence of the experimental conditions on the features of the resulting Cs-AuNPs, the reaction parameters were varied, for the first time, as follows: chitosan with four different molecular weight (VLM_w_, LM_w_, MM_w_ and HM_w_), two different chitosan concentrations (0.1 and 0.4 *w*/*v* %), two different Au concentrations (0.13 and 0.26 mM) and three reaction solvents (acetic acid, acetic acid/methanol and acetic acid/isopropanol). In all the experiments, the first observable indication of Cs-AuNPs formation was a color change in the reaction mixture from transparent/pale yellow to pale red/wine red/purplish after 30 min of reaction. Similar color changes were observed in previous works [6,13,18,58]. The appearance of a red color in the reaction solution is due to the excitation of surface plasmon resonance in the AuNPs. The chitosan acts both as a reducing and stabilizing agent, i.e., the chitosan reduces the Au(III) ions in the AuCl_4−_ ions to Au(0), thus forming the AuNPs. According to Prema and Thangapandiyan [58], the electrostatic forces between the amino groups of chitosan (positively charged) and the surface of the AuNPs (negatively charged) led to the synthesis of Cs-AuNPs of high stability. 

### 3.1. Influence of Chitosan Molecular Weight on the Physical Features of Cs-AuNPs

Four types of chitosan with different molecular weights (from VLM_w_ to HM_w_) were used to reduce the Au(III) ions in order to ascertain whether if molecular weight is a determinant factor in the synthesis of Cs-AuNPs. However, it should be noted that only three are really comparable (LM_w_, MM_w_ and HM_w_), since the VLM_w_ has an average molecular weight that is within the size distribution of the LM_w_ and, in addition, VLM_w_ has a higher degree of deacetylation than the others. The color of the Cs-AuNP suspensions can be compared in Figure S1 of the Supplementary Materials. From the UV-vis spectra shown in Figure S2 of the Supplementary Materials and their analysis, it could be inferred that, in general terms, a lower chitosan molecular weight (which implies shorter molecular chains) leads to faster crystalline growth. Similar results were found by C. Sun et al. [14].

For comparison purposes, Table 2 shows the hydrodynamic diameter (expressed as Z-average), the polydispersity index (PdI), the zeta potential and the electrophoretic mobility of the Cs-AuNPs synthesized in experiments 1 to 8. From the results, it can be inferred that the size of the Cs-AuNPs is influenced by the chitosan concentration more by chitosan molecular weight. The PdI values were very similar within each series but slightly higher when chitosan concentration was 0.4% (around 0.5), meaning that higher chitosan concentrations will result in Cs-AuNPs with a wider size distribution. Similar size and PdI values were found for biosynthesized Cs-AuNPs in previous works [6]. In the case of the zeta potential and electrophoretic mobility, positive values (around 50 mV and 4 µm·cm/Vs, respectively) were found in all cases (as was expected, given the positive charge of chitosan), but no clear trend that depended on chitosan molecular weight could be established. The positive surface charge of Cs-AuNPs is provided by the protonation of the amino groups (-NH_3_^+^) of chitosan in acid medium, which are presumably on the surface of the AuNPs [13]. The zeta potential values are higher than those found in previous works [6] reflecting higher stability and preventing Cs-AuNP aggregation because of the sufficiently high repulsive forces between the individual particles [59]. These results indicate that chitosan is a good stabilizing agent for AuNPs.

### 3.2. Influence of the Chitosan Concentration on the Biosynthesis of Cs-AuNPs

Two chitosan concentrations (0.1 and 0.4 *w*/*v* %) were chosen for the reduction of Au(III) ions to see whether the chitosan concentration is a significant parameter in the synthesis of Cs-AuNPs. The results are shown in Table 1. The analysis has been completed in the Supplementary Materials. From the UV-vis spectra shown in Figure S3 of the Supplementary Materials, it could be concluded that AuNP synthesis by means of this process seems to be greatly influenced by the Cs:Au molar ratio. However, it should be noted that the refractive index of polymeric stabilizing agents may influence the band of surface plasmons.

The effect of chitosan concentration on the physical parameters of the resulting Cs-AuNPs was also established (see Table 2). It is clear that when the chitosan concentration increases for a given chitosan with a fixed molecular weight, the Cs-AuNP diameter greatly increases, which is similar to the conclusion reached by Abrica-González et al. [60]. In the same way, the PdI also increases, leading to a wider size distribution. As regards the zeta potential, the higher chitosan concentration led to a higher value of this parameter and higher electrophoretic mobility values in all cases.

### 3.3. Influence of the Gold Concentration on the Biosynthesis of Cs-AuNPs

Two different Au(III) concentrations (0.13 and 0.26 mM) were used to analyze the influence of this parameter on the synthesis of Cs-AuNPs using MM_w_ chitosan and a fixed chitosan concentration (0.1 or 0.4 *w*/*v* %). Therefore, experiment 3 can be compared with experiment 9 and experiment 7 with experiment 12 (see Table 3 and Figure S4 of the Supplementary Materials). In both cases, the Cs:Au molar ratio was halved. The results showed in Figure S5 of the Supplementary Materials confirm those found in Section 3.2, namely that the Cs:Au molar ratio is determinant for the progress of the reduction reaction due, among other factors, to the viscosity of the reaction mixture. According to C. Sun et al. [14], the chitosan chain is broken during the reaction, which implies a decrease in molecular weight and the viscosity of the solution. This effect becomes more pronounced as the Cs:Au molar ratio decreases.

As regards the size of the resulting Cs-AuNPs (Table 3), it can be seen that, for a fixed MM_w_ chitosan concentration, the Z-average and the PdI values decrease when the gold concentration increases. Therefore, it seems that low Cs:Au molar ratios lead to small Cs-AuNPs and a narrower size distribution. These results also confirm those found in Section 3.2. Regarding the zeta potential and electrophoretic mobility, we conclude their values do not change significantly with variations in the gold concentration.

### 3.4. Influence of the Reaction Solvent on the Biosynthesis of Cs-AuNPs

Apart from using acetic acid as reaction solvent, we used mixtures of acetic acid/aliphatic alcohols in order to evaluate whether these alcohols (methanol and isopropanol) contribute to a higher yield of Cs-AuNPs or they are even smaller. For this purpose, experiments 3−10, 12−13 and 9−11 can be independently compared (see Figure S6 of the Supplementary Materials). From the analysis of UV-vis spectra showed in Figure S7 of the Supplementary Materials, we found again that the Cs:Au molar ratio is a key parameter in Cs-AuNP synthesis, so it is possible that, when this parameter reaches a certain value, e.g., with a high proportion of chitosan (such as an 80:1 molar ratio), the reaction yield decreases independently of the reaction solvent used.

The physical parameters of the Cs-AuNPs synthesized in the experiments described in this section are presented in Table 4. As can be seen, the inclusion of an alcohol in the reaction solvent system led to larger Cs-AuNPs in all cases compared to the use of acetic acid alone. The increase in the hydrodynamic diameter was especially noticeable in the sample obtained with isopropanol. In the case of zeta potential and electrophoretic mobility, the incorporation of an alcohol led to significantly lower values, this difference again being more pronounced in the samples synthesized in acetic acid/isopropanol mixtures.

### 3.5. Transmission Electron Microscopy

**Figure 2:**
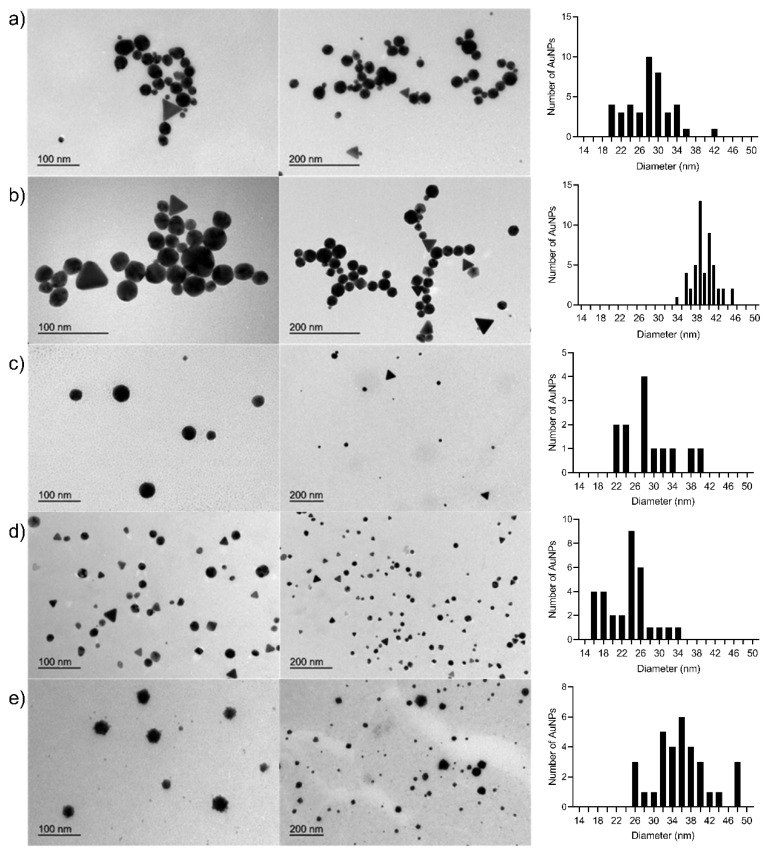
TEM images and size distributions of Cs-AuNPs prepared in experiments (**a**) 3, (**b**) 4, (**c**) 11, (**d**) 12 and (**e**) 13.

Figure 2 shows the TEM images of some of the Cs-AuNPs biosynthesized in the experiments carried out in this study. When the samples of experiments 3, 4, 11, 12 and 13 were analyzed, the Cs-AuNPs were generally spherical in shape, but some polygonal nanocrystals were also observed. Similar morphologies for Cs-AuNPs have been previously reported [54,61].

The TEM images showed the size of the Au cores to be: 27.98 ± 4.91 nm (experiment 3), 27.67 ± 5.01 nm (experiment 4), 28.93 ± 5.98 nm (experiment 11), 23.08 ± 4.50 nm (experiment 12), 36.81 ± 7.61 nm (experiment 13). In the Supplementary Materials, these values obtained from TEM are compared with the estimated values of the AuNP diameter based on the UV-vis spectra and the values obtained from DLS measurements (see Table S4 of the Supplementary Materials). In addition, we have obtained TEM images of the Cs-AuNPs (experiment 4) with and without staining using uranyl acetate to obtain additional information on the morphology of the coating layer of chitosan. Figure S8 shows TEM pictures of the Cs-AuNPs (experiment 4) with no staining. The observed size and shape of the Cs-AuNPs are in agreement with the previous discussion. Figure S9 shows TEM pictures of the Cs-AuNPs (experiment 4) subjected to a staining procedure using uranyl acetate. The morphology of the chitosan layer is mainly spherical and distributed homogeneously over the surface of the AuNPs.

### 3.6. Antibacterial Activity

The next step was to evaluate the antibacterial activity of the 13 Cs-AuNPs reported here. It should be noted that the structural characteristics of the Cs-AuNPs were modified in order to evaluate how such changes may influence their antibacterial activity. Among the modified variables, the most important were: the concentration of chitosan, its molecular weight and size of the AuNPs. The antibacterial activities of the different Cs-AuNPs were evaluated against the most common resistant bacterial pathogen MSSA, MRSA and EC strains and CI-EC clinical isolate from infections of patients from the public hospital. The lowest concentration of Cs-AuNPs to inhibit the growth of the bacteria was considered as MIC, while the lowest concentration that allowed no growth after sub culturing from MIC was regarded as MBC.

Results are shown in Table 5. Thirteen Cs-AuNPs were evaluated, three of them (Experiments 6, 7 and 8, marked in bold in Table 5) displayed significant antibacterial activity. It was shown that the Cs-AuNPs obtained in these experiments inhibited the normal growth of strains MSSA, MRSA and EC with MIC values between 16 and 32.5 µg/mL. While the MBC were about 2-fold higher than the final MIC (32.5 µg/mL) to experiment 6 and 7 against MSSA and CI-EC respectively, equal MBC values to their MIC (i.e., 32.5 and 65 µg/mL) were obtained in the experiments 6−8 against all bacteria assayed.

As regards CI-EC, this clinical isolate was more sensitive to Cs-AuNPs of the experiment 7, with MIC values of 16.2 µg/mL, similar to the selective effect shown by Cs-AuNPs of the experiment 6 against MSSA. All the results depicted in Table 5 could be observed after six hours of the test. The results illustrate how the antibacterial activity of these Cs-AuNPs correlates very well with five physical parameters: chitosan concentration, gold concentration, Cs:Au molar ratio, Z-average and zeta potential. These five parameters showed the following ranges in all the active Cs-AuNPs: Au Concentration (0.4 *w*/*v* %), Cs:Au molar ratio (160:1), Z-average (175−194 nm) and zeta potential (49−75 mV). It appears that these structural characteristics must coincide for the biological response to be displayed; however, a critical size above 150 nm and a zeta potential of around 50 mV would be necessary to produce a significant level of antibacterial activity against to bacteria of clinical importance.

Cs-AuNPs of the experiments 1−4 showed significant differences as regards these physical parameters: gold concentration (0.1 *w*/*v* %), Cs:Au molar ratio (40:1), Z-average (49−54 nm) and zeta potential (44−48 mV); whereas in the case of system 5 the differences might be observed in the values of Z-average (98 nm) and zeta potential (49 mV). None of these Cs-AuNPs showed antibacterial effects. These results are in a complete agreement with the nonspecific mechanism that we have previously proposed for antimicrobial peptides called Dynamic Action Mechanism (DAM) [48,62]. This mechanism postulates that, in a first phase, interactions between the Cs-AuNPs and the bacterial cell membrane take place through electrostatic interactions between both charged species. Thus, an adequate charge density in the Cs-AuNPs will favor interactions between Cs-AuNPs and the bacterial membrane. This allows suitable interactions between the Cs-AuNPs and the membranes, followed by a structural modification and loss of the properties of the membrane. In order to visualize this mechanism in more detail, molecular dynamics simulations were carried out, which are discussed in the next section.

### 3.7. Simulation Results

**Figure 3:**
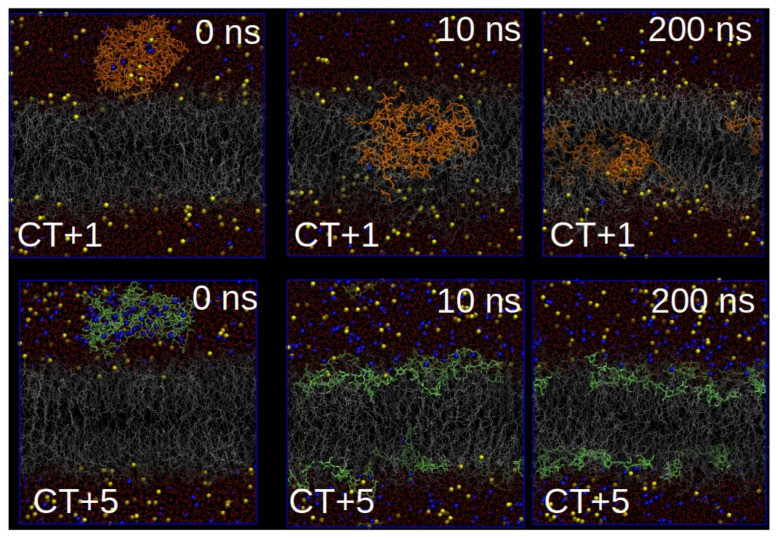
Cartoon corresponding to different snapshots related with the interaction of the two different models of aggregates of chitosan with different charge, CT+1 and CT+5. Red points: water, yellow beads: Na+, blue beads: CL-, Orange bonds: CT+1, green bonds: CT+5, gray lines: DPPC and silver–gray lines: DPPS.

Figure 3 shows different snapshots for both systems corresponding to CT+1 and CT+5 along the simulated trajectories. In the system corresponding to CT+1, chitosan was seen to penetrate into the lipid bilayer after 10 ns of simulation time, which produced important perturbations in the structure of the lipid bilayer, as can be observed from the variations in bilayer thickness in the presence of the chitosan aggregate. This behavior contrasts with that observed in the system corresponding to CT+5, in which, due to the high charge density of chitosan, they disperse on the lipid bilayer due to the strong electrostatic interactions between CT+5 and the charged surface on the lipid bilayer. Almost no protrusion is observed on the lipid bilayer and, as a consequence, the structure of the lipid bilayer remains unperturbed.

These results are in line with the antimicrobial activity of the Cs-AuNPs, in which charge density is a critical parameter for their antibacterial activity, a property that has been associated with the strong electrostatic interactions of chitosan with the charge surface of the lipid bilayer of bacterial cell membranes. In this regard, we expect that the action mechanism of these Cs-AuNPs follows a non-specific action mechanism, such as that described by López Cascales et al. [48] for the DAM of small cationic antibacterial peptides. In summary, our simulations show how the surface charge density of chitosan aggregates (see Table S1 of the Supplementary Materials) is a critical parameter for controlling antibacterial activity when they are adsorbed onto AuNPs.

## 4. Conclusions

AuNPs were successfully biosynthesized using chitosan as both the stabilizing and reducing agent. The size distributions of the AuNPs and Cs-AuNPs were 15−56 nm and 44−345 nm, respectively. The values found for the zeta potential were positive and sufficiently high to ensure good stability and to prevent Cs-AuNP aggregation because of the high repulsive forces between the individual particles. The results showed that the size of the Cs-AuNPs is influenced to a greater extent by the chitosan concentration than by the chitosan molecular weight. It seems that when the concentration increases for a chitosan of a fixed molecular weight, the Cs-AuNP diameter greatly increases. In the case of the zeta potential, a higher chitosan concentration leads to a higher zeta potential in all cases. On the other hand, the incorporation of an alcohol in the reaction solvent system leads to the production of larger Cs-AuNPs and significantly lowers the zeta potential in all cases, particularly when isopropanol is used.

Regarding the antibacterial activity, the Cs-AuNPs obtained displayed important antibacterial activity when the chitosan concentration increases for a given chitosan with a fixed molecular weight, affecting the normal growth of the ATCC strains MSSA, MRSA and EC, with MIC values of between 16 and 32.5 µg/mL. CI-EC was more sensitive to Cs-AuNPs of the experiment with MM_w_ chitosan (MIC = 16.2 µg/mL), and a similar selective effect was presented by Cs-AuNPs of the experiment with LM_w_ against MSSA. We conclude that some of the structural characteristics must be complimented all together in order to display the biological response: Au Concentration (0.4 *w*/*v* %), Cs:Au molar ratio (160:1), Z-average (175−194 nm) and zeta potential (49−75 mV); however, a critical size above 150 nm and a zeta potential of around 50 mV would is necessary to produce a significant antibacterial effect against to bacteria of clinical importance. The results of the simulations are in line with the antimicrobial activity of the Cs-AuNPs, in which charge density is a critical parameter for their antibacterial activity. This property has been associated with strong electrostatic interactions of chitosan with the charge surface of the lipid bilayer of bacterial cell membranes, suggesting that the action mechanism of these Cs-AuNPs follows a non-specific action mechanism. Data obtained in this work suggest that Cs-AuNPs are promising nanostructures for reducing bacterial infections, respecting the integrity of mammalian cells, and displaying selectivity against studied bacteria.

## Figures and Tables

**Table 1:** Summary of the experiments performed to obtain Cs-AuNPs and their experimental parameters.

Experiment	Cs Molecular Weight	Cs Concentration (*w*/*v* %)	Au Concentration (mM)	Solvent	Cs:Au Molar Ratio	pH
1	VLM_w_	0.1	0.13	Acetic acid	40:1	3.22
2	LM_w_	0.1	0.13	Acetic acid	40:1	3.01
3	MM_w_	0.1	0.13	Acetic acid	40:1	2.95
4	HM_w_	0.1	0.13	Acetic acid	40:1	2.97
5	VLM_w_	0.4	0.13	Acetic acid	160:1	3.41
6	LM_w_	0.4	0.13	Acetic acid	160:1	3.45
7	MM_w_	0.4	0.13	Acetic acid	160:1	3.48
8	HM_w_	0.4	0.13	Acetic acid	160:1	3.44
9	MM_w_	0.1	0.26	Acetic acid	20:1	2.98
10	MM_w_	0.1	0.13	Acetic acid/methanol	40:1	3.41
11	MM_w_	0.1	0.26	acetic acid/isopropanol	20:1	3.36
12	MM_w_	0.4	0.26	Acetic acid	80:1	3.41
13	MM_w_	0.4	0.26	Acetic acid/methanol	80:1	4.13

VLM_w_: Very low molecular weight; LM_w_: Low molecular weight; MM_w_: Medium molecular weight; HM_w_: High molecular weight.

**Table 2:** Physical parameters of the Cs-AuNPs synthesized in the experiments 1 to 8.

Exp.	Cs Concentration (*w*/*v* %)	Cs:Au Molar Ratio	Cs Molec Weight	Z-Average (nm)	Zeta Potential (mV)	PdI	Electrophoretic Mobility (µm·cm/Vs)
1	0.1	40:1	VLM_W_	64.02 ± 0.14 ^a^	46.3 ± 2.25 ^ac^	0.317 ± 0.006 ^a^	3.632 ± 0.176 ^ab^
2			LM_W_	49.48 ± 0.14 ^b^	44.83 ± 1.47 ^a^	0.334 ± 0.002 ^a^	3.516 ± 0.112 ^b^
3			MM_W_	52.57 ± 1.11 ^b^	46.1 ± 1.06 ^ac^	0.315 ± 0.005 ^a^	3.613 ± 0.082 ^ab^
4			HM_W_	54.86 ± 0.08 ^b^	48.66 ± 2.11 ^ac^	0.319 ± 0.004 ^a^	3.812 ± 0.166 ^ab^
5	0.4	160:1	VLM_W_	98.1 ± 3.76 ^c^	49.53 ± 3.48 ^abc^	0.491 ± 0.039 ^b^	3.881 ± 0.273 ^abc^
6			LM_W_	185.13 ± 7.12 ^d^	55.23 ± 2.14 ^bd^	0.486 ± 0.023 ^b^	4.328 ± 0.166 ^c^
7			MM_W_	175.76 ± 2.97 ^e^	49.76 ± 2.4 ^ad^	0.494 ± 0.006 ^b^	3.902 ± 0.185 ^abc^
8			HM_W_	194 ± 2.08 ^d^	51.3 ± 1.99 ^cd^	0.456 ± 0.002 ^b^	4.020 ± 0.158 ^ac^

VLM_w_: Very low molecular weight; LM_w_: Low molecular weight; MM_w_: Medium molecular weight; HM_w_: High molecular weight. Mean ± standard deviation (SD) (*n* = 3); ^a,b,c,d,e^ Means within each column with different letters are significantly different (*p* < 0.05), Tukey’s test.

**Table 3:** Physical parameters of the Cs-AuNPs synthesized with MM_w_ chitosan in experiments 3−9 and 7−12.

Exp.	Cs Concentration (*w*/*v* %)	Au Concentration (mM)	Cs:Au Molar Ratio	Z-Average (nm)	Zeta Potential (mV)	PdI	Electrophoretic Mobility (µm·cm/Vs)
3	0.1	0.13	40:1	52.57 ± 1.11 ^a^	46.1 ± 1.06 ^a^	0.315 ± 0.005 ^a^	3.613 ± 0.082 ^a^
9		0.26	20:1	44.21 ± 0.51 ^b^	46.5 ± 2 ^a^	0.309 ± 0.008 ^a^	3.646 ± 0.158 ^a^
7	0.4	0.13	160:1	175.76 ± 2.97 ^c^	49.76 ± 2.4 ^a^	0.494 ± 0.006 ^b^	3.902 ± 0.185 ^a^
12		0.26	80:1	112 ± 1.65 ^d^	48.4 ± 3.27 ^a^	0.339 ± 0.037 ^a^	3.792 ± 0.257 ^a^

Mean ± standard deviation (SD) (*n* = 3). ^a,b,c,d,^ Means within each column with different letters are significantly different (*p* < 0.05), Tukey’s test.

**Table 4:** Physical parameters of the Cs-AuNPs synthesized with MM_w_ chitosan in the experiments 3-10, 12−13 and 9−11.

Experiments	Cs Concentration (*w*/*v* %)	Au Concentration (mM)	Cs:Au Molar Ratio	Reaction Solvent	Z-Average (nm)	Zeta Potential (mV)	PdI	Electrophoretic Mobility (µm·cm/Vs)
3	0.1	0.13	40:1	Acetic acid	52.57 ± 1.11 ^a^	46.1 ± 1.06 ^a^	0.315 ± 0.005 ^a^	3.613 ± 0.082 ^a^
10				Acetic acid/methanol	81.76 ± 0.87 ^b^	24.9 ± 1.70 ^b^	0.550 ± 0.006 ^b^	1.950 ± 0.132 ^b^
12	0.4	0.26	80:1	Acetic acid	112 ± 1.65 ^c^	48.4 ± 3.27 ^a^	0.339 ± 0.037 ^a^	3.792 ± 0.257 ^a^
13				Acetic acid/methanol	345 ± 1.1 ^d^	27.6 ± 1.59 ^b^	0.347 ± 0.043 ^a^	2.179 ± 0.124 ^b^
9	0.1	0.26	20:1	Acetic acid	44.21 ± 0.50 ^e^	46.5 ± 2 ^a^	0.309 ± 0.008 ^a^	3.646 ± 0.158 ^a^
11				Acetic acid/isopropanol	301 ± 4.85 ^f^	11.4 ± 0.2 ^c^	0.206 ± 0.017 ^c^	0.893 ± 0.017 ^c^

Mean ± standard deviation (SD) (*n* = 3). ^a,b,c,d,e,f^ Means within each column with different letters are significantly different (*p* < 0.05), Tukey’s test.

**Table 5:** Antibacterial activity of the synthesized Cs-AuNPs. Tests were made in triplicate and MIC values are expressed as µg/mL.

Bacteria		Cs-AuNPs													
		1	2	3	4	5	6	7	8	9	10	11	12	13	Cef
**Gram (+)**															
MSSA	MIC	65	65	65	>65	32.5	**16.2**	**32.5**	**32.5**	>65	>65	65	65	65	0.50
	MBC	>65	>65	>65	-	65	**32.5**	**32.5**	**32.5**	-	-	65	65	65	0.80
MRSA	MIC	65	32.5	>65	>65	65	**32.5**	**32.5**	**32.5**	>65	>65	65	65	65	0.50
	MBC	>65	65	-	-	-	**32.5**	**32.5**	**32.5**	-	-	65	65	65	0.50
**Gram (−)**															
EC	MIC	65	65	>65	>65	65	**65**	**65**	**65**	>65	>65	>65	65	65	1.9
	MBC	65	>65	-	-		**65**	**65**	**65**	-	-	-	65	65	2.5
CI-EC	MIC	65	65	65	65	32.5	**32.5**	**16.2**	**32.5**	>65	>65	65	32.5	65	1
	MBC	>65	>65	>65	>65	32.5	**32.5**	**32.5**	**32.5**	-	-	>65	65	>65	1

Cef: Cefatoxime; MSSA: methicillin-sensitive *Staphylococcus aureus* ATCC 25923; MRSA: methicillin-resistant *Staphylococcus aureus* ATCC 43300; EC: *Escherichia coli* ATCC 25922; CI-EC: Clinical isolate *Escherichia coli* 11046; MIC: minimum inhibitory concentration; MBC: minimum bactericidal concentration.

## Supplementary Materials

The following are available online at https://www.mdpi.com/2079-4991/10/12/2340/s1, Figure S1: Cs-AuNP dispersions after synthesis using VLM_w_, LM_w_, MM_w_ or HM_w_ at 0.1 or 0.4 *w*/*v* % chitosan concentration, Figure S2: UV-vis absorption spectra for AuNPs as a function of the chitosan molecular weight at a chitosan concentration of 0.1% and 0.4% *w*/*v*, Figure S3: UV-vis absorption spectra for AuNPs formation as a function of the chitosan concentration for VLM_w_, LM_w_, MM_w_ and HM_w_, Figure S4: Cs-AuNPs dispersions after synthesis with MM_w_ chitosan and different gold concentrations (0.13 and 0.26 mM). (a) 0.1 *w*/*v* % and (b) 0.4 *w*/*v* % chitosan concentration, Figure S5: UV-vis absorption spectra for AuNPs formation with MM_w_ chitosan as a function of the gold concentration. (a) 0.1 *w*/*v* % and (b) 0.4 *w*/*v* % chitosan concentration, Figure S6: Cs-AuNP dispersions after synthesis with MM_w_ chitosan and different reaction solvent mixtures. (a) 0.1 *w*/*v* % chitosan concentration and 0.13 mM of gold concentration; (b) 0.4 *w*/*v* % chitosan concentration and 0.26 mM gold concentration; (c) 0.1 *w*/*v* % chitosan concentration and 0.26 mM gold concentration, Figure S7: UV-vis absorption spectra for AuNPs formation with MM_w_ chitosan as a function of the reaction solvent system (acetic acid, acetic acid/methanol 50 *v*/*v* % or acetic acid/isopropanol 50 *v*/*v* %). (a) 0.1 *w*/*v* % chitosan concentration and 0.13 mM gold concentration; (b) 0.4 *w*/*v* % chitosan concentration and 0.26 mM gold concentration; (c) 0.1 *w*/*v* % chitosan concentration and 0.26 mM gold concentration. Table S1: Surface charge density (C/m^2^). Table S2: List of parameters used to calculate the surface charge density. Table S3: Charge distribution of CT+1 and CT+5. The atom numeration of this table correlates with the atom numeration of Figure 1, Table S4: Diameter of AuNPs obtained from UV-vis spectra and comparison with the values of size obtained from DLS measurements and from TEM.
