# Supplementary material for: Antibacterial Effect of Chitosan–Gold Nanoparticles and Computational Modeling of the Interaction between Chitosan and a Lipid Bilayer Model
Source: Nanomaterials (Basel). 2020 Nov 25;10(12):2340. doi: 10.3390/nano10122340 (PMC7761461; doi:10.3390/nano10122340)
Supplement: Supplementary file 1 [file nanomaterials-10-02340-s001.pdf]

nanomaterials

SUPPLEMENTARY MATERIALS

Antibacterial Effect of Chitosan–Gold Nanoparticles and Computational Modeling of the Interaction between Chitosan and a Lipid Bilayer Model

M. G. Fuster [1], M. G. Montalbán [1,*], G. Carissimi [1], B. Lima [2,3], G. E. Feresin [2,3], M. Cano [4], J. J. Giner-Casares [4], J. J. López-Cascales [5], R. D. Enriz [6] and G. Víllora [1]

[1] Chemical Engineering Department, Faculty of Chemistry, University of Murcia, 30100 Murcia, Spain; marta.g.f@um.es (M.G.F.); guzmanaugusto.carissimin@um.es (G.C.); gvillora@um.es (G.V.)
[2] Instituto de Biotecnología, Universidad Nacional de San Juan, Av. Libertador General San Martín 1109 (O), San Juan CP 5400, Argentina; blima@unsj.edu.ar (B.L.); gferesin@unsj.edu.ar (G.E.F.)
[3] CONICET (Consejo Nacional de Ciencia y Tecnología), CABA, Buenos Aires C1405DJR, Argentina
[4] Departamento de Química Física y Termodinámica Aplicada, Instituto Universitario de Nanoquímica (IUNAN), Facultad de Ciencias, Universidad de Córdoba, Campus de Rabanales, Ed. Marie Curie, E-14071 Córdoba, Spain; q82calum@uco.es (M.C.); jjginer@uco.es (J.J.G.-C.)
[5] Dep. Ing. Química y Ambiental, Campus Alfonso XIII, Universidad Politécnica de Cartagena, Aulario C, Cartagena, 30203 Murcia, Spain; javier.lopez@upct.es
[6] Facultad de Química, Bioquímica y Farmacia, Instituto Multidisciplinario de Investigaciones Biológicas (IMIBIO-SL), CONICET, Universidad Nacional de San Luis, Ejército de los Andes 950, San Luis 5700, Argentina; denriz@unsl.edu.ar
* Correspondence: mercedes.garcia@um.es

Surface charge density calculation

The surface charge density (σ) of a spherical colloidal particle can be obtained following the method proposed by Makino & Ohshima [1] on the basis of the zeta potential and the ionic strength of the medium. For a nanoparticle with constant surface charge density, the surface charge density is a more characteristic quantity than the zeta potential, ψ_0, because for such particles the zeta potential is not a constant and depends on the electrolyte concentration. The method is based on Equation (1):

$$\sigma = \frac{2\varepsilon_r\varepsilon_0\kappa kT}{ze}\sinh\left(\frac{ze\psi_0}{2kT}\right)\left[1+\frac{1}{\kappa a}\frac{1}{cosh^2(ze\psi_0/4kT)}+\frac{1}{(\kappa a)^2}\frac{8\ln\left[\cosh\left(\frac{ze\psi_0}{4kT}\right)\right.}{sinh^2(ze\psi_0/2kT)}\right]^{1/2} \quad (1)$$

where k is the Boltzmann constant, T is the absolute temperature, $\mathcal{E}_r$ is relative permittivity, $\mathcal{E}_0$ is the permittivity of the vacuum, $\mathcal{E}$ is the permittivity, N_A is the Avogadro's number, e is the elementary electric charge, I is the Ionic strength; a is the radius of the spherical nanoparticles, Ψ_0 is the zeta potential, z is the electrolyte valence and where κ is the Debye-Hückel parameter defined by Equation (2):

$$\kappa = \left[\frac{1000e^2N_A(2I)}{\varepsilon kT}\right]^{1/2} \quad (2)$$

The results of the calculated surface charge density for each experiment have been included in Table S1 and all the parameter values used for the calculation are shown in Table S2.

Table S1. Surface charge density (C/m²).

Exp.	Surface Charge Density (C/m²)
1	5.12 ± 0.24·10⁻²
2	4.95 ± 0.16·10⁻²
3	5.12 ± 0.20·10⁻²
4	5.47 ± 0.21·10⁻²
5	5.54 ± 0.38·10⁻²
6	6.37 ± 0.25·10⁻²
7	5.55 ± 0.26·10⁻²
8	5.77 ± 0.22·10⁻²
9	5.19 ± 0.21·10⁻²
10	2.51 ± 0.17·10⁻²
11	1.10 ± 0.18·10⁻²
12	5.38 ± 0.32·10⁻²
13	2.77 ± 0.19·10⁻²

Mean ± standard deviation (SD) (*n*=3).

Table S2. List of parameters used to calculate the surface charge density.

Parameter	Value	Units
Relative permittivity 25 °C, ε_r	78.30	*Dimensionless*
Permittivity of a vacuum, ε_0	$8.85 \cdot 10^{-12}$	$C^2/(J \cdot m)$
Permittivity, ε	$6.93 \cdot 10^{-10}$	$C^2/(J \cdot m)$
Boltzmann constant, k	$1.38 \cdot 10^{-23}$	J/K
Avogadro's number, N_A	$6.02 \cdot 10^{23}$	mol^{-1}
Elementary electric charge, e	$1.60 \cdot 10^{-19}$	C
Temperature, T	298.15	K
Ionic strength, I	$1.74 \cdot 10^{-1}$	M
Debye-Hückel parameter, κ	$1.04 \cdot 10^{8}$	m^{-1}
Electrolyte valence, z	1	*Dimensionless*

UV-vis Spectroscopy

UV-vis absorbance spectra of the synthesized Cs-AuNPs were recorded on a Thermo Spectronic Heλios α spectrophotometer (Thermo Fisher Scientific, Waltham, MA, USA) in the wavelength range of 750-450 nm. The spectra were obtained for preliminary confirmation of AuNP formation from the surface plasmon resonance band around 530 nm [2]. One milliliter of sample and a quartz cell were used for the measurements and double deionized water as blank. The diameter, d, of the AuNPs obtained in each experiment was estimated from UV-vis parameters using Equation (3), as in Haiss et al. [3]:

$$d = exp\left[B_1 \frac{A_{spr}}{A_{450}} - B_2\right] \quad (3)$$

where A_{spr} is the absorbance at the surface plasmon resonance peak, A_{450} is the absorbance at λ = 450 nm and B_1 and B_2 are empirical parameters with values of 3.00 and 2.20, respectively.

Table S3. Charge distribution of CT+1 and CT+5. The atom numeration of this table correlates with the atom numeration of Figure 1.

	CT+1		CT+5	
Atom Number	Atom Type	Charge (e)	Atom Type	Charge (e)
1	CH1	0,028	CH1	0,14
2	CH1	0,0242	CH1	0,121
3	CH1	0,03	CH1	0,15
4	O	-0,0472	O	-0,236
5	CH1	0,0257	CH1	0,128
6	CH1	0,0214	CH1	0,107
7	OS	-0,0502	OS	-0,251
8	H	0,041	H	0,205
9	N	0,13	N	0,65
10	CH2	0,0268	CH2	0,134
11	O	-0,0522	O	-0,261
12	H	0,034	H	0,17
13	O	-0,0474	O	-0,237
14	H	0,035	H	0,175
15	CH2	0,024	CH2	0,12
16	O	-0,0418	O	-0,209
17	CH2	0,0284	CH2	0,142
18	CH1	0,036	CH1	0,18
19	CH1	0,0218	CH1	0,109
20	CH1	0,028	CH1	0,14
21	O	-0,045	O	-0,225
22	CH1	0,03	CH1	0,15
23	CH1	-0,0012	CH1	-0,006
24	O	-0,0558	O	-0,279
25	H	0,0415	H	0,207
26	N	0,124	N	0,62
27	CH2	0,0252	CH2	0,126
28	O	-0,0486	O	-0,243
29	H	0,0332	H	0,166
30	CH2	0,024	CH2	0,12
31	O	-0,0414	O	-0,207
32	CH2	0,0274	CH2	0,137
33	CH1	0,036	CH1	0,18
34	CH1	0,022	CH1	0,11
35	CH1	0,0283	CH1	0,141
36	O	-0,0444	O	-0,222
37	CH1	0,0296	CH1	0,148
38	CH1	-0,0008	CH1	-0,004
39	O	-0,056	O	-0,28
40	H	0,0402	H	0,201
41	N	0,126	N	0,63
42	CH2	0,0252	CH2	0,126
43	O	-0,0488	O	-0,244
44	H	0,033	H	0,165
45	CH2	0,0236	CH2	0,118
46	O	-0,0412	O	-0,206
47	CH2	0,0272	CH2	0,136
48	CH1	0,0348	CH1	0,174
49	CH1	0,022	CH1	0,11
50	CH1	0,0284	CH1	0,142
51	O	-0,0438	O	-0,219
52	CH1	0,0292	CH1	0,146

Influence of chitosan molecular weight on the physical features of Cs-AuNPs

The color of the resulting dispersion after Cs-AuNPs formation was observed to depend on the chitosan molecular weight (Figure S1). The color of AuNP solutions can be red, pink, violet, purple, blue or yellow with increasing particle size and the formation of aggregates [4].

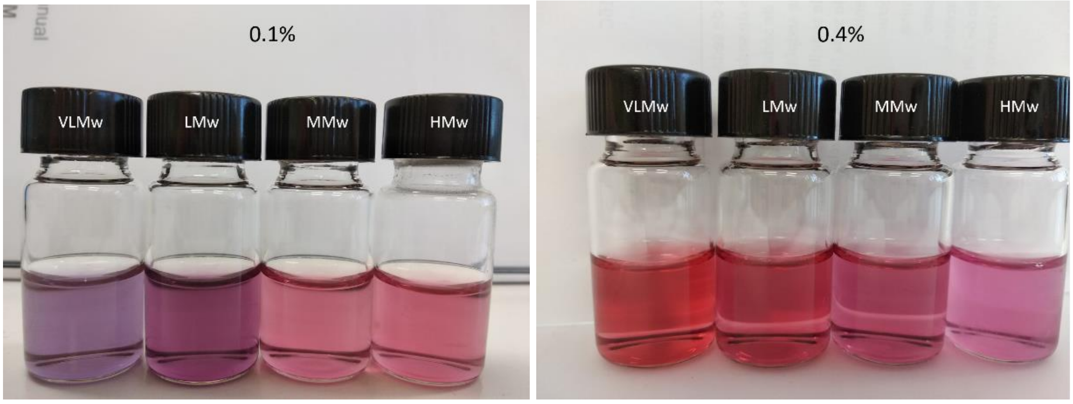

Figure S1. Cs-AuNP dispersions after synthesis using VLMw, LMw, MMw or HMw at 0.1 or 0.4 w/v % chitosan concentration.

The formation and resulting stability of AuNPs can be followed by UV-vis spectroscopy. AuNPs usually absorb light in the visible range depending on their size, local refractive index and aggregation leading to plasmon coupling. For bigger AuNPs, the plasmon absorption band is shifted to longer wavelengths [5]. The intensity of the absorption band is also sensitive to the size and shape of the AuNPs, the increasing intensity of the absorption maximum corresponding to greater AuNP formation growth [6]. Figure S2 shows the UV-vis spectra of the Cs-AuNPs synthesized from chitosan of different molecular weights. The spectra have been normalized with respect to the absorbance at the lowest wavelength. The absorption bands of the Cs-AuNPs were usually observed at around 530 nm, which is indicative of AuNP formation [7]. Only the peak of the spectra of VLM_w at 0.1% and of HM_w at 0.4% chitosan concentrations were displaced to longer wavelengths of 564 nm and 538 nm, respectively. The shift to 538 nm was attributed to a local change in the refractive index provoked by the chitosan coating, while the shoulder at ca. 570 nm might correspond to small aggregates of AuNPs within a shell of chitosan. It was also seen that the intensity of the absorption increased as the molecular weight decreased (when the concentration of CS was 0.4%), indicating a higher degree of reduction of the metal ions. This trend was not so evident for the lower chitosan concentration.

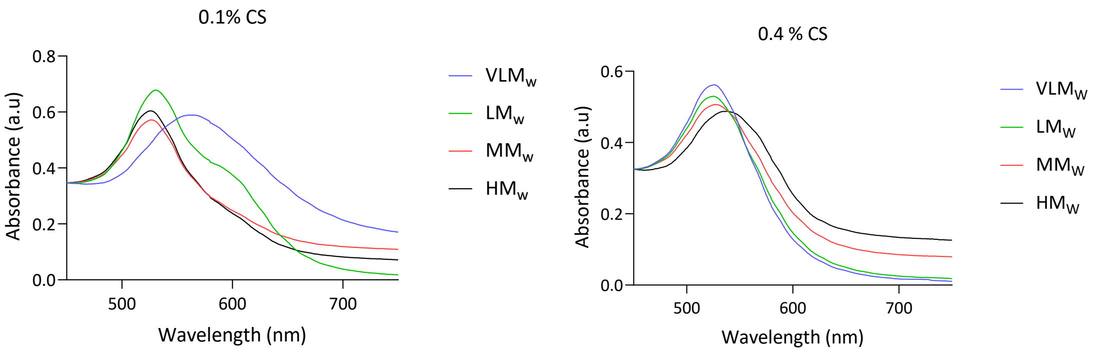

Figure S2. UV-vis absorption spectra for AuNPs as a function of the chitosan molecular weight at a chitosan concentration of 0.1% and 0.4% w/v.

Influence of the chitosan concentration on the biosynthesis of Cs-AuNPs

As can be seen in Figure S1, the color of the colloid after Cs-AuNP formation follows two different trends as a function of the concentration of chitosan. In the case of 0.1 w/v % chitosan, VLMW and LMW led to purple colloidal dispersions, indicating aggregation of the AuNPs. This aggregation is further confirmed by the UV-vis data (see Figure S2). With greater molecular weights of chitosan, the extent of aggregation was reduced and AuNPs appeared as individual nanoparticles rather than aggregates. Therefore, for a 0.1 w/v % of chitosan, there is a threshold molecular weight below which the polymer chains do not provide sufficient steric stabilization and a modest aggregation occurs. By contrast, the aggregation of the AuNPs was much more difficult for 0.4 w/v % chitosan and individual unaggregated AuNPs were found for the entire range of molecular weight (see Figure S2). Only for the highest molecular weight was a small shift observed, probably corresponding to a local variation in the refractive index, as might be expected from the rather bulky polymer shell.

Figure S3 shows the UV-vis spectra of the Cs-AuNPs synthesized from different chitosan concentrations. As can be seen, for LM_w, MM_w and HM_w chitosans, the higher concentration (0.4 w/v %), i.e. higher Cs:Au molar ratio, led to a lower rate of AuNP synthesis. This may have been due to the increased viscosity, which controls the nucleation and growth of AuNPs [8]. These results agree with those of other authors who claimed that, as the gold precursor concentration increases, i.e. lower Cs:Au molar ratio, the plasmon peak shows greater absorbance, indicating greater progress in the gold reduction process [9].

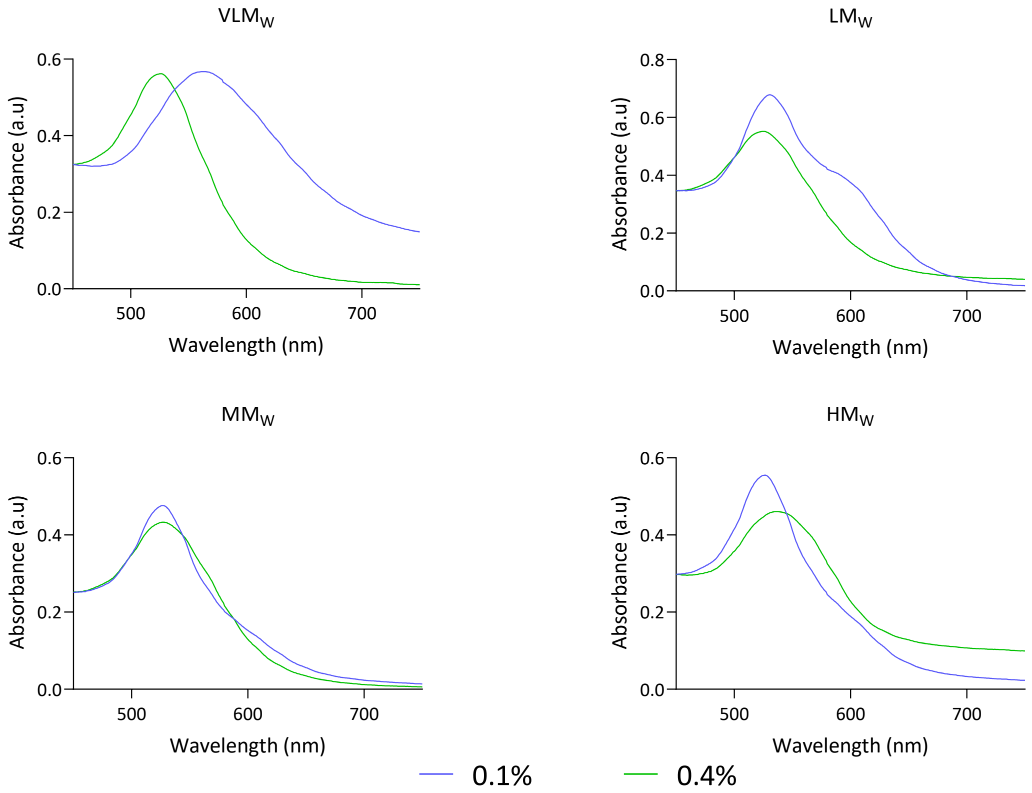

Figure S3. UV-vis absorption spectra for AuNPs formation as a function of the chitosan concentration for VLMw, LMw, MMw and HMw.

Influence of the gold concentration on the biosynthesis of Cs-AuNPs

As regards the color of the resultant colloids (Figure S4), an increase in the concentration of gold led to a more intense color, as was to be expected from the higher amount of AuNPs formed. The same conclusion was reached by Regiel-Futyra et al. [9].

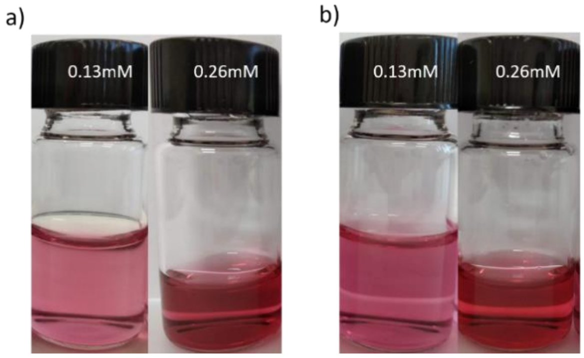

Figure S4. Cs-AuNPs dispersions after synthesis with MMw chitosan and different gold concentrations (0.13 and 0.26mM). **a**) 0.1 w/v % and **b**) 0.4 w/v % chitosan concentration.

Figure S5 shows the UV-vis absorption spectra of the AuNPs obtained as a function of the gold concentration. As can be seen, an increase in the gold concentration clearly contributed to a higher rate of reduction and hence a higher AuNP synthesis; see the increase in peak absorbance in the plasmon absorption band (around 530 nm).

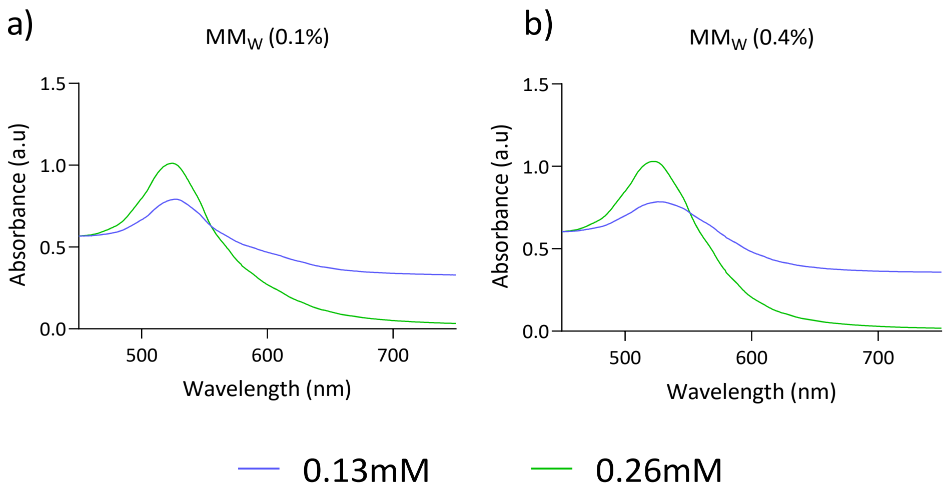

Figure S5. UV-vis absorption spectra for AuNPs formation with MMw chitosan as a function of the gold concentration. **a**) 0.1 w/v % and **b**) 0.4 w/v % chitosan concentration.

Influence of the reaction solvent on the biosynthesis of Cs-AuNPs

The pairs of experiments 3–10 and 12–13 are useful for determining the effect of methanol and experiments 9–11 for determining the effect of isopropanol. It is important to remark that in all cases the alcohol was incorporated in the reaction mixture in a proportion of 50% volume, the rest being acetic acid. Figure S6 shows the differences in the color of the colloids obtained. At a first glance, it seems that the presence of alcohols in the reaction mixture contribute to making the solution cloudier, suggesting an enhanced interaction with the chitosan coating and leading to the formation of micron-size aggregates.

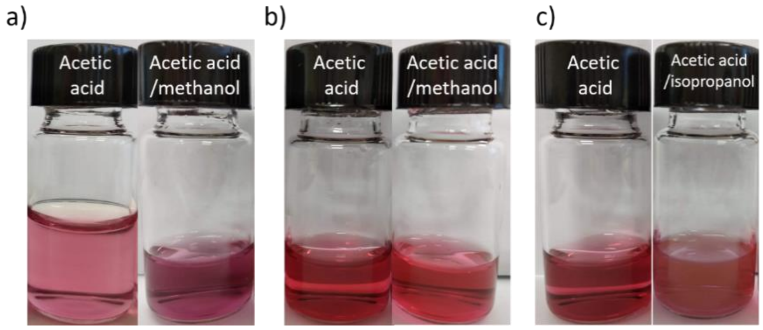

Figure S6. Cs-AuNP dispersions after synthesis with MMw chitosan and different reaction solvent mixtures. **a**) 0.1 w/v % chitosan concentration and 0.13 mM of gold concentration; **b**) 0.4 w/v % chitosan concentration and 0.26 mM gold concentration; **c**) 0.1 w/v % chitosan concentration and 0.26 mM gold concentration.

Figure S7 shows the UV-vis absorption spectra for AuNP formation with MM_w chitosan as a function of the reaction solvent system (acetic acid, acetic acid/methanol 50 v/v % or acetic acid/isopropanol 50 v/v %). Based on the synthesis parameters used for the experiments of Figures S7a and S7c, we can assume that the alcohols, especially methanol, do not contribute to improving the Cs-AuNP yield that can be obtained using acetic acid alone because their spectra show a lower degree of intensity at maximum absorbance. However, in the case of Figure S7b, methanol seems to slightly improve Cs-AuNP synthesis. This discrepancy is probably due to the different Cs:Au molar ratios used. In the experiments of Figures S7a and S7c, the molar ratios were lower, 40:1 and 20:1, respectively, but in the experiments of Figure S7b a molar ratio of 80:1 was used.

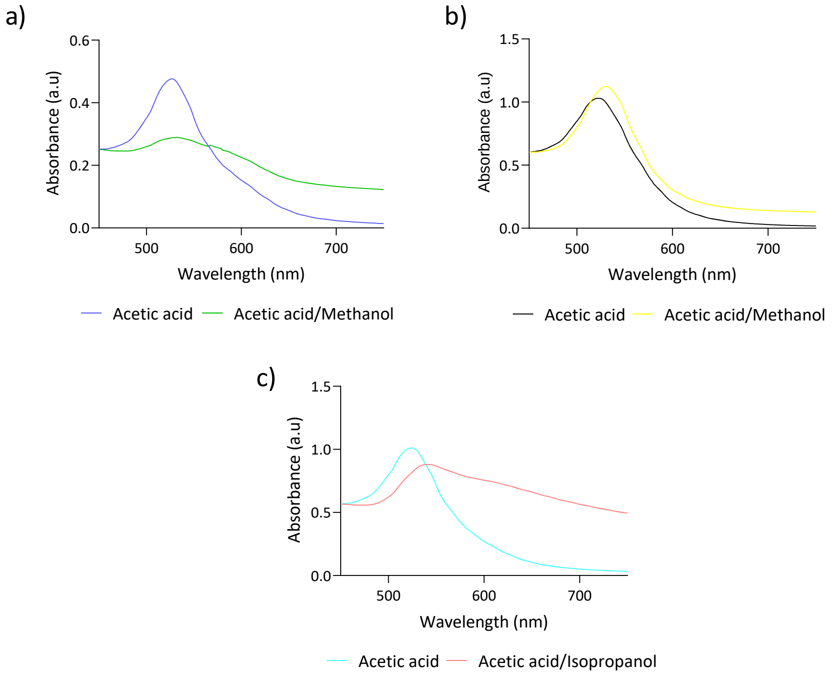

Figure S7. UV-vis absorption spectra for AuNPs formation with MMw chitosan as a function of the reaction solvent system (acetic acid, acetic acid/methanol 50 v/v % or acetic acid/isopropanol 50 v/v %). **a**) 0.1 w/v % chitosan concentration and 0.13 mM gold concentration; **b**) 0.4 w/v % chitosan concentration and 0.26 mM gold concentration; **c**) 0.1 w/v % chitosan concentration and 0.26 mM gold concentration.

Estimation of size and concentration of AuNPs from UV-vis spectra

The size of AuNPs is an important variable for any proposed medical or industrial application. In this case, the size of Cs-AuNPs, i.e. the whole entity formed by the core and the polymer shell, should be differentiated from the size of the AuNP itself (the core). The location of the absorption band in the UV-vis spectra provides approximate information on the core size as does TEM, but the total size of the core-shell particle is equally important. The latter can be assessed by DLS and hydrodynamic diameter measurements. Table S4 shows the estimated values of the diameter based on the UV-vis spectra using Equation (3), and the values obtained from DLS measurements and from TEM. Note that DLS gives the hydrodynamic diameter, which includes the AuNP, the chitosan coating and the water of the hydration sphere, while TEM only provides the size of the AuNP, meaning that these values should be significantly lower than those obtained by DLS.

Table S4. Diameter of AuNPs obtained from UV-vis spectra and comparison with the values of size obtained from DLS measurements and from TEM.

Exp.	Cs Molecular Weight	Cs Conc. (w/v %)	Au Conc. (mM)	Solvent	Cs:Au Molar Ratio	*d* (nm) from UV-vis Spectra (3)	*d* (nm) From DLS	*d* (nm) From TEM
**1**	VLM_w	0.1	0.13	Acetic acid	40:1	48.87	64.02 ± 0.14	-
**2**	LM_w	0.1	0.13	Acetic acid	40:1	39.09	49.48 ± 0.14	-
**3**	MM_w	0.1	0.13	Acetic acid	40:1	32.47	52.57 ± 1.11	27.98 ± 4.91
**4**	HM_w	0.1	0.13	Acetic acid	40:1	29.62	54.86 ± 0.08	27.67 ± 5.01
**5**	VLM_w	0.4	0.13	Acetic acid	160:1	19.84	98.10 ± 3.76	-
**6**	LM_w	0.4	0.13	Acetic acid	160:1	15.27	185.13 ± 7.12	-
**7**	MM_w	0.4	0.13	Acetic acid	160:1	18.58	175.76 ± 2.97	-
**8**	HM_w	0.4	0.13	Acetic acid	160:1	22.88	194 ± 2.08	-
**9**	MM_w	0.1	0.26	Acetic acid	20:1	23.34	44.21 ± 0.50	-
**10**	MM_w	0.1	0.13	Acetic acid/ Methanol	40:1	22	81.76 ± 0.87	-
**11**	MM_w	0.1	0.26	Acetic acid/ Isopropanol	20:1	15.50	301 ± 4.85	28.93 ± 5.98
**12**	MM_w	0.4	0.26	Acetic acid	80:1	18.53	112 ± 1.65	23.08 ± 4.50
**13**	MM_w	0.4	0.26	Acetic acid/ Methanol	80:1	56.16	345 ± 1.10	36.81 ± 7.61

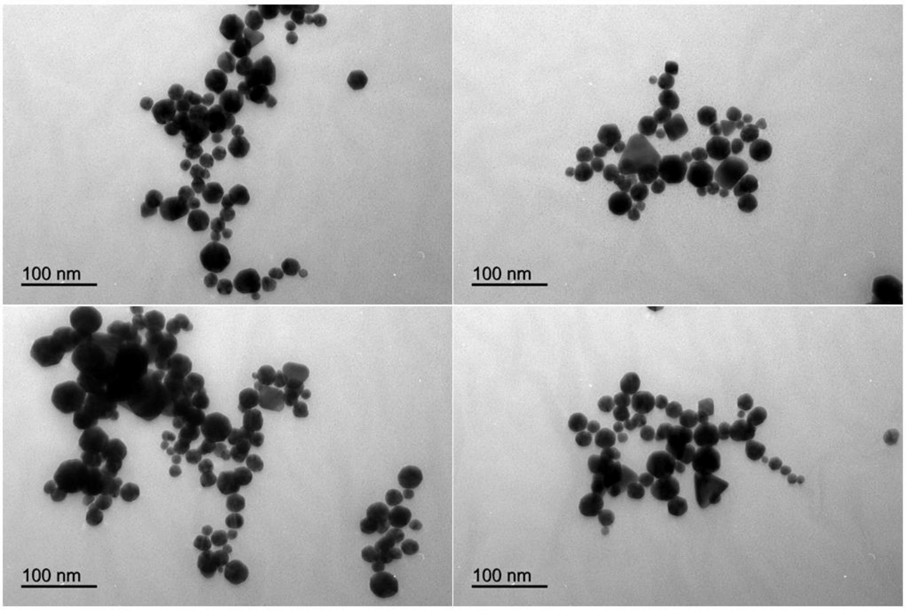

Figure S8. TEM pictures of experiment 4. Scale bar in the inset.

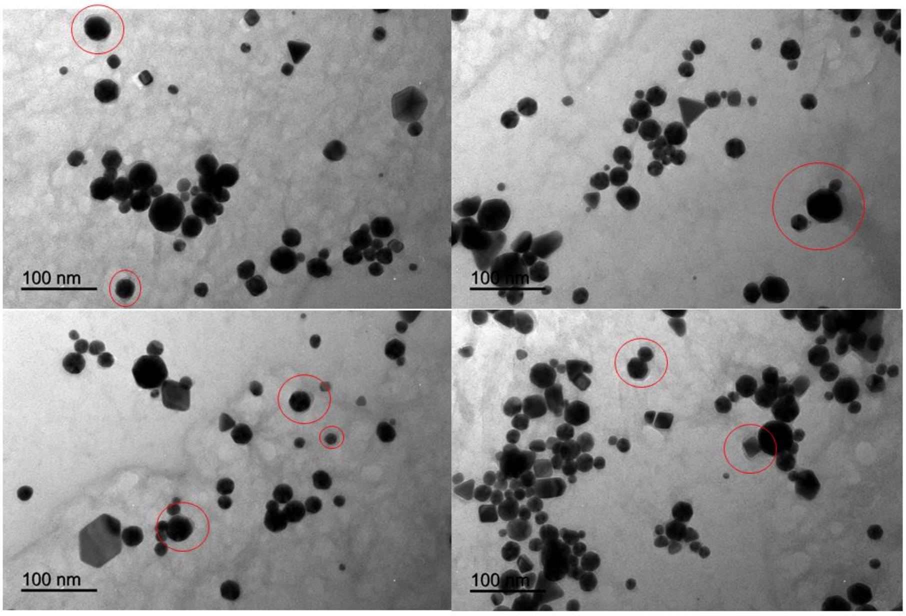

Figure S9. TEM pictures of sample 4 after staining with uranyl acetate. Scale bar in the inset. Red circles are included for highlighting representative nanoparticles.

References:

1. Makino, K.; Ohshima, H. Electrophoretic mobility of a colloidal particle with constant surface charge density. *Langmuir* **2010**, *26*, 18016–18019.
2. Abrica-González, P.; Zamora-Justo, J.A.; Sotelo-López, A.; Vázquez-Martínez, G.R.; Balderas-López, J.A.; Muñoz-Diosdado, A.; Ibáñez-Hernández, M. Gold nanoparticles with chitosan, N-acylated chitosan, and chitosan oligosaccharide as DNA carriers. *Nanoscale Res. Lett.* **2019**, *14*, 258.
3. Haiss, W.; Thanh, N.T.K.; Aveyard, J.; Fernig, D.G. Determination of Size and Concentration of Gold Nanoparticles from UV−Vis Spectra. *Anal. Chem.* **2007**, *79*, 4215–4221.
4. Kim, J.-Y.; Lee, J.-S. Synthesis and Thermally Reversible Assembly of DNA−Gold Nanoparticle Cluster Conjugates. *Nano Lett.* **2009**, *9*, 4564–4569.
5. Czechowska-Biskup, R.; Rokita, B.; Ulański, P.; Rosiak, J.M. Preparation of gold nanoparticles stabilized by chitosan using irradiation and sonication methods. *Prog. Chem. Appl. Chitin Deriv*. **2015**, *20*, 18-33.
6. Simeonova, S.; Georgiev, P.; Exner, K.S.; Mihaylov, L.; Nihtianova, D.; Koynov, K.; Balashev, K. Kinetic study of gold nanoparticles synthesized in the presence of chitosan and citric acid. *Colloid. Surface. A* **2018**, *557*, 106-115.
7. Katas, H.; Lim, C.S.; Nor Azlan, A.Y.H.; Buang, F.; Mh Busra, M.F. Antibacterial activity of biosynthesized gold nanoparticles using biomolecules from Lignosus rhinocerotis and chitosan. *Saudi Pharm. J.* **2019**, *27(2)*, 283–292.
8. Pestov, A.; Nazirov, A.; Modin, E.; Mironenko, A.; Bratskaya, S. Mechanism of Au(III) reduction by chitosan: Comprehensive study with 13C and 1H NMR analysis of chitosan degradation products. *Carbohyd. Polym*. **2015**, *117*, 70–77.
9. Regiel-Futyra, A.; Kus-Liśkiewicz, M.; Sebastian, V.; Irusta, S.; Arruebo, M.; Stochel, G.; Kyzioł, A. Development of noncytotoxic chitosan-gold nanocomposites as efficient antibacterial materials. *ACS Appl. Mater. Inter.* **2015**, *7(2)*, 1087–1099.
